# Integrating Pharmacological Features to Personalized Models With Statin Therapy: A Glimpse Into the Future?∗

**DOI:** 10.1016/j.jacadv.2024.100892

**Published:** 2024-03-06

**Authors:** Eliano P. Navarese, Marta Casula, Gavino Casu

**Affiliations:** Departments of Clinical Experimental Cardiology, Clinical Interventional Cardiology, University of Sassari, Sassari, Sardinia Island, Italy

**Keywords:** personalized, pharmacological features, statin therapy

**Figure:**
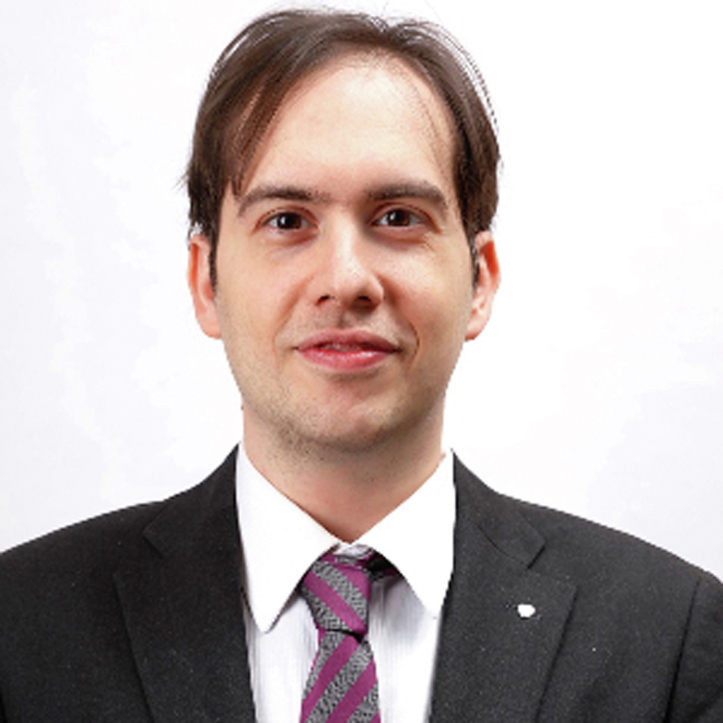

Statin therapy's effectiveness can vary significantly among individuals, influenced by genetic, environmental, and clinical factors. Studies have shown genetic variations can significantly impact statin metabolism and efficacy.^1^^,^^2^ Additionally, clinical factors like age, gender, race, and comorbidities also contribute to this variability. These findings highlight the complexity of statin response as well as the inefficiency of one-size-fits-all approach to treat patients. Residual risk remains still high despite potent statin therapy,^3^ which might be attributable to variations in pharmacodynamic and pharmacokinetic of statins, in turn highlighting the need for personalized approaches to improve patient outcomes.

The study by Aggarwal et al^4^ published in this issue of *JACC: Advances* examined the individualized dose-response to statins and its association with cardiovascular disease outcomes. It focused on the variable response to a variety of statins (atorvastatin, simvastatin, or rosuvastatin) among patients, modeled using pharmacological properties like baseline low-density lipoprotein cholesterol (E0), potency (ED50), and maximum low-density lipoprotein cholesterol reduction (Emax). The authors leveraged electronic health record data to analyze the relationship between statin potency and efficacy with real-world cardiovascular outcomes.

Findings from the current report are compelling in suggesting that ED50 and Emax are significant predictors of atherosclerotic cardiovascular disease events (ischemic heart disease, cerebrovascular disease, peripheral vascular disease, and coronary artery revascularization procedure) and mortality at a long-term follow-up of 10 years. Results remained consistent across different types of statins.

In contrast, there was no association with a polygenic risk score for coronary artery disease. Thus, while ED50 and Emax may more closely reflect variability in drug response genes, these parameters were not associated with variability in disease genes.

In this framework, these pharmacological parameters may serve as a guiding tool in evaluating the potency and efficacy of statins once integrated into new equations to assess individual responses to statin therapy. Integrating pharmacological modeling with patient data, the study offers new insights into personalized medicine, specifically in cardiovascular disease management, and represents a significant step toward more effective, individualized treatment strategies.

By leveraging these new equations, which allow to derive phenotypic traits according to dose-response relation, clinicians can potentially predict and enhance the efficacy and safety of statin therapy for individual patients, leading to more effective and personalized treatment regimens.

The novelty of the study lies in its unique focus on modeling individual responses to statin therapy using pharmacological parameters and its application of these models to real-world health data. This approach contrasts with traditional, more generalized statin therapy strategies conventionally applied.

The study's introduction of new equations for tailoring statin therapies is thus a potentially significant innovation. These equations may allow for a more nuanced understanding of how individual patients metabolize and respond to statins, taking into account specific pharmacological properties. By enabling a more personalized prediction of statin efficacy and safety, these equations might lead to more effective and safer statin therapy tailored to individual patient needs.

However, the current study did not address whether these equations might serve to safely treat patients by reducing statin toxicity, which remains another potential new research frontier that could be addressed. Balancing efficacy and toxicity risk trade-off elaborating “*net benefit*” equations could enhance the statin compliance without compromising efficacy, ultimately leading to a further lowering of residual risk and hard endpoints as well as minimizing statin side effects.

The study examined various statins, but it is unclear whether the observed effects are class effects. This is because the study lacked the power to analyze the effects of individual statins, especially rosuvastatin, which had a smaller sample size compared to other statin groups. Moreover, it remains uncertain whether these findings might be replicated in multicenter cohorts, which is essential for generalizability of results.

In conclusion, the findings from the study could significantly influence future clinical practices in statin therapy management. Personalizing statin doses using individual biomarkers and tailored equations can enhance treatment effectiveness and minimize side effects. In this context, a global approach focused globally on the *net benefits* of pharmacological interventions could revolutionize the management of cardiovascular diseases. Aligning treatments more accurately with each patient's unique physiological response promises to improve outcomes and patient satisfaction in statin therapy. However, additional research is necessary to create innovative net benefit equations and polygenic risk scores that incorporate drug response genes. This advancement will usher in a new era of personalized lipid-lowering therapy that balances benefits and risks and is specifically tailored to individual drug responses.

## Funding support and author disclosures

The authors have reported that they have no relationships relevant to the contents of this paper to disclose.
